# Staphylococcal decolonization to prevent surgical site infection: Is there a role in colorectal surgery?

**DOI:** 10.1017/ash.2022.262

**Published:** 2022-07-11

**Authors:** Rasha Raslan, Michelle Doll, Heather Albert, Hirsh Shah, Jaime Bohl, Kaila Cooper, Michael P. Stevens, Gonzalo Bearman

**Affiliations:** 1 Virginia Commonwealth University Medical Center, Richmond, Virginia; 2 Virginia Commonwealth University School of Medicine, Richmond, Virginia

## Abstract

**Objective:**

We implemented a preoperative staphylococcal decolonization protocol for colorectal surgeries if efforts to further reduce surgical site infections (SSIs).

**Design:**

Retrospective observational study.

**Setting:**

Tertiary-care, academic medical center.

**Patients:**

Adult patients who underwent colorectal surgery, as defined by National Healthcare Safety Network (NHSN), between July 2015 and June 2020. Emergent cases were excluded.

**Methods:**

Simple and multivariable logistic regression were performed to evaluate the relationship between decolonization and subsequent SSI. Other predictive variables included age, sex, body mass index, procedure duration, American Society of Anesthesiology (ASA) score, diabetes, smoking, and surgical oncology service.

**Results:**

In total, 1,683 patients underwent nonemergent NHSN-defined colorectal surgery, and 33.7% underwent the staphylococcal decolonization protocol. SSI occurred in 92 (5.5%); 53 were organ-space infections and 39 were superficial wound infections. We detected no difference in overall SSIs between those decolonized and not decolonized (*P* = .17). However, superficial wound infections were reduced in the group that received decolonization versus those that did not: 7 (1.2%) of 568 versus 32 (2.9%) of 1,115 (*P* = .04).

**Conclusions:**

Staphylococcal decolonization may prevent a subset of SSIs in patients undergoing colorectal surgery.

Surgical site infections (SSIs) are associated with significant morbidity and mortality, prolonged length of hospitalization and increased healthcare costs.^1,2^ Colorectal surgery is associated with a high risk of surgical site infection (SSI), with reported incidence as high as 16%–20%.^2^ However, the national burden of colorectal SSI, as reported to the National Healthcare Safety Network (NHSN) from acute-care hospitals between 2016 and 2018, was consistently 2.3% of all colorectal surgeries.^3^ Many institutions have introduced bundled interventions that successfully reduced the risk of postoperative SSI.^4,5^ In an era of decreasing SSI rates, there is intense interest in additional interventions to drive infection rates ever closer to zero.

Staphylococcal decolonization protocols attempt to alter the microbiome to prevent staphylococcal and other skin flora from accessing the surgical site. Decolonization is effective in reducing SSIs in orthopedic, cardiothoracic, and neurosurgical procedures^6,7^; however, it is not a traditional component of the SSI prevention bundle in general or colorectal surgery.^8^

*Staphylococcus aureus* is a common cause of SSI, and colorectal surgeries in the modern era may be no exception, particularly with regard to infections acquired in the surgical wound.^8^ Furthermore, chlorhexidine gluconate (CHG) has good activity against not only gram-positive organisms, such as *Staphylococcus aureus*, but also has activity against gram-negative bacteria and *Candida* spp.^6^ There may be a missed opportunity for manipulation of the skin microbiome to prevent SSI in the colorectal surgery population.

A staphylococcal decolonization protocol was enacted for colorectal surgeries at our institution beginning in August 2017; this study compares patient outcomes between patients who did and did not undergo preoperative staphylococcal decolonization.

## Methods

This study was performed at an 865-bed tertiary-care hospital. All colorectal surgeries from July 2015 to June 2020 were reviewed for SSI as defined by NHSN criteria.^9^ Surveillance for colorectal surgeries for NHSN is a prospective process in which the chart of each patient with an NHSN colorectal surgical procedure is manually reviewed for evidence of SSI occurring in the 30 days following the procedure, and outside records are included in this process when available. In August 2017, a decolonization protocol was developed with input from colorectal surgery, anesthesia, pharmacy, infection prevention and nursing stakeholders. Staphylococcal decolonization was performed for 5 days prior to surgery using 2% CHG bodywash solution daily, and mupirocin nasal ointment and 0.12% CHG oral rinse, both twice daily. Prior to 2017, individual providers decolonized their patients with the same protocol at their discretion. Patient compliance with the protocol was documented in the chart on the day of surgery. To validate the accuracy of the documentation, the study team matched archived patient-completed decolonization checklists against the documentation in the medical record. Thus, compliance was determined by both patient report and provider documentation.

Colorectal SSIs were diagnosed using NHSN criteria. The primary outcome was SSI, and secondary outcomes were superficial wound infection (SWI), deep wound infection (DWI), and organ-space or intra-abdominal infection (IAI). Predictive variables included decolonization status (yes or no), age, sex, body mass index, procedure duration, American Society of Anesthesiology (ASA) score, diabetes, smoking, and surgical oncology service.

Preferred surgical antimicrobial prophylaxis with cefazolin and metronidazole or cefoxitin (with the addition of vancomycin if the patient was known to be MRSA colonized), and CHG skin preparation were standard throughout the study period. Emergent cases were excluded from the analysis. No preoperative screening for MSRA or other specific organisms was performed.

Baseline characteristics of decolonized versus nondecolonized surgical populations were compared using the χ^2^ or the Student *t* test. Simple and multivariable logistic regression were performed to evaluate the relationship between decolonization and subsequent SSI and between decolonization and subsequent SWI. Multivariable models were controlled for all clinically important variables above. A second model controlled for only those variables found to be statistically significant in the regression analysis predicting SSI. All statistical analyses were performed using SAS version 9.4 software (SAS Institute, Cary, NC). Our protocol was approved by the VCU Institutional Review Board.

## Results

In total 2,141 patients underwent colorectal surgery during the study period, and 1,683 patients underwent nonemergent NHSN-defined colorectal surgery from July 2015 to June 2020. Among them, 736 cases were at least partially laparoscopic (43.7%), and 568 patients (33.7%) completed the decolonization protocol. Characteristics of decolonized versus nondecolonized patients are shown in Table 1. The proportion of patients completing decolonization ranged from 17.5% in 2017 to 47.0% in 2020; proportions completing decolonization by year are shown in Appendix 1.

**Table 1.** Patient Characteristics

Variable	Staphylococcal Decolonization (N=568)	No Staphylococcal Decolonization (N=1115)	*P* Value
Age, mean y (95% CI)	56.5 (55.2–57.7)	56.5 (55.5–57.5)	.9723
BMI, mean (95% CI)	28.5 (27.9–29.1)	27.9 (27.4–28.3)	.0890
**Sex, No. (%)**			
Male	291 (51.2)	634 (56.9)	.0282
Female	277 (48.7)	481 (43.1)	
**ASA score, no. (%)**			
1	4 (0.7)	14 (1.3)	.0086
2	169 (29.8)	323 (29.0)	
3	358 (63.0)	651 (58.4)	
4	37 (6.5)	127 (11.4)	
**Diabetes mellitus, no. (%)**			
Yes	68 (12.0)	172 (15.4)	.1106
No	478 (84.2)	910 (81.6)	
Unknown	22 (3.8)	33 (3.0)	
**Smoking, no. (%)**			
Never	260 (45.8)	413 (37.0)	<.0001
Former	183 (32.2)	383 (34.4)	
Current	114 (20.1)	222 (19.9)	
Unknown	11 (1.9)	97 (8.7)	
Duration, mean min (95% CI)	216 (205–227)	191 (184–197)	<.0001
**Closure, no. (%)**			
Primary	522 (91.9)	1009 (90.5)	.0134
Other	18 (3.2)	68 (6.1)	
Unknown	28 (4.9)	38 (3.4)	
**Wound class, no. (%)**			
Clean	18 (3.2)	37 (3.3)	.8500
Clean-contaminated	508 (89.4)	983 (88.2)	
Contaminated	39 (6.9)	85 (7.6)	
Dirty/infected	3 (0.5)	9 (0.8)	

Furthermore, 92 patients (5.5%) developed an SSI: 53 were IAIs and 39 were SWIs. There were no deep wound infections. We detected no difference in overall SSIs between those decolonized and not decolonized (*P* = .17) in unadjusted analysis; the SSI rate in the decolonized group was 4.4% (25 of 568) versus 6.0% (67 of 1,115) in the group that was not decolonized. However, SWIs were reduced in the group that received decolonization versus those who did not: 7 (1.2%) of 568 versus 32 (2.9%) of 1,115 (*P* = .04). Decolonization was the only significant predicting variable for SWI in unadjusted analysis, and overall SSI was better predicted by age (odds ratio [OR], 1.02; 95% confidence interval [CI], 1.00–1.03; *P* = .03) and past or present tobacco use (*P* = .02). We detected no difference in the rate organ-space infections (IAIs) in those decolonized versus not: 3.2% (18 of 568) vs 3.1% (35 of 1,115), respectively (*P* = .97) in unadjusted analysis (Appendix 2). None of the other variables were significantly associated with SSI or SWI in unadjusted analyses.

When adjusting for known SSI risk factors, those receiving decolonization remained at decreased risk for SWIs with an odds ratio of 0.38 (95% CI, 0.15–0.92; *P* =.03) (Table 3). Overall SSIs (both SWIs and IAIs) continued to be better predicted by age (*P =* .03) and past or present tobacco use (*P* = .04) in the adjusted analysis (Table 2, model 2). Body mass index (BMI) became a significant predictor in adjusted analysis for SWI only (*P* = .04), not for overall SSI (*P =* .44).

**Table 2.** Predictors of Surgical Site Infection (SSI)

Variable	Unadjusted OR (95% CI)	*P* Value	Adjusted OR (95% CI) Model 1	*P* Value	Adjusted OR (95% CI) Model 2	*P* Value
Staphylococcal decolonization (ref, no)	0.720 (0.450–1.153)	.1720	1.488 (0.903–2.452)	.1188	1.551 (0.951–2.529)	.0790
**Age**	**1.016 (1.002–1.030)**	**.0267**	**1.022 (1.006–1.038)**	**.0085**	**1.016 (1.001–1.031)**	**.0324**
Body mass index	1.004 (0.975–1.033)	.7968	1.013 (0.980–1.047)	.4401	…	…
Sex (ref, female)	1.069 (0.700–1.634)	.7571	0.933 (0.592–1.469)	.7635	…	…
ASA score (ref, ≤2)					…	…
3 or 4	0.848 (0.543–1.323)	.4669	0.712 (0.434–1.170)	.1805		
Diabetes mellitus (ref, no)	1.003 (0.547–1.837)	.9933	0.859 (0.454–1.626)	.6410	…	…
**Smoking (ref, never)**						**.0443**
Former	**1.942 (1.192–3.162)**	**.0189**	1.875 (1.124–3.128)	.0548	**1.839 (1.127–3.002)**	
Current	**1.152 (0.614–2.160)**		1.425 (0.741–2.741)		**1.229 (0.651–2.320)**	
Duration of procedure	1.001 (0.999–1.002)	.2534	1.001 (0.999–1.003)	.2091	…	…
Closure (ref, primary)	1.292 (0.548–3.049)	.5580	1.359 (0.516–3.580)	.5351	…	…
**Wound class (ref, C/CC)**					…	…
Contaminated/dirty	0.502 (0.181–1.389)	0.1846	0.600 (0.213–1.689)	0.3329		

Note. OR, odds ratio; CI, confidence interval; ref, reference, ASA, American Society of Anesthesiology; C/CC, clean/clean-contaminated. Bold indicates statistical significance.

**Table 3.** Predictors of Superficial Wound Infection (SWI)

Variable	Unadjusted OR (95% CI)	*P* Value	Adjusted OR (95% CI) Model 1	*P* Value	Adjusted OR (95% CI) Model 2	*P* Value
**Staphylococcal decolonization (ref, no)**	**0.422 (0.185–0.963)**	**.0404**	**0.376 (0.154–0.917)**	**.0315**	**0.358 (0.148–0.866)**	**.0226**
Age	1.009 (0.989–1.030)	.3748	1.015 (0.992–1.039)	.2118	1.008 (0.986–1.029)	1.029
Body mass index	1.030 (0.991–1.070)	.1394	**1.048 (1.003–1.095)**	**.0366**	…	…
Sex (ref, female)	1.062 (0.560–2.015)	.8540	0.874 (0.439–1.739)	.7012	…	…
**ASA Score (ref, 1–2)**					…	…
3–4	0.771 (0.398–1.497)	.4428	0.625 (0.299–1.130)	.2136		
Diabetes mellitus (ref, no)	0.675 (0.237–1.920)	.4611	0.545 (0.183–1.628)	.2770	…	…
**Smoking (ref, never)**						.1500
Former Current	2.303 (1.062–4.994) 1.617 (0.632–4.136)	.1062	2.112 (0.954–4.674) 1.838 (0.697–4.845)	.1743	2.164 (0.995–4.704) 1.622 (0.628–4.189)	
Duration of procedure	0.998 (0.94–1.001)	.1465	0.998 (0.994–1.001)	.1615	…	…
Closure (ref, primary)	2.086 (0.724–6.008)	.1732	1.695 (0.481–5.980)	.4118	…	…
**Wound class (ref, C/CC)**					…	…
Contaminated/dirty	0.294 (0.040–2.158)	.2287	0.312 (0.042–2.330)	.2560		

Note. OR, odds ratio; CI, confidence interval; ref, reference, ASA, American Society of Anesthesiology; C/CC, clean/clean-contaminated. Bold indicates statistical significance.

Of 92 SSIs, 44 (47.8%) had organisms identified on culture: 32 of 53 IAIs and 12 of 39 SWIs. Also, IAIs infections were more likely to have 1 or more microbial diagnoses. Organisms recovered from SWIs included *Staphylococcus aureus* (n = 2), *Staphylococcus lugdunensis* (n = 1), *Enterobacteriacae* spp (n = 4), *Candida* spp (n = 2), *Enterococcus faecalis* (n = 2), *Bacteroides fragilis* (n = 1), *Pseudomonas aeruginosa* (n = 1), *Acinetobacter baumanii* (n = 1). Organisms recovered from IAIs included *Enterobacteriacae* spp (n = 16), *Enterococcus* spp (n = 7), *Candida* spp (n = 6), other skin flora (coagulase-negative *Staphylococcus*, *Bacillus* spp, *Ruminococcus*; n = 4), *Bacteroides fragilis* (n = 4), *Pasteurella multocida* (n = 1), *Pseudomonas aeruginosa* (n = 2), *Streptococcus bovis* (n = 1), *Staphylococcus aureus* (n = 1), and *Clostridium* spp (n = 1). The 3 patients with *Staphylococcus aureus* as a cause of SSI did not undergo the decolonization protocol, and 2 of the 3 isolates were methicillin-resistant (MRSA).

## Discussion

Preoperative staphylococcal decolonization is routinely utilized for nongeneral surgeries including orthopedic, cardiac, and neurosurgical cases, particularly if implants are involved because existing literature has suggested benefits in SSI reduction.^6^ However, bundled approaches to SSI reduction touch many aspects of perioperative care, and decolonization protocols impacting *Staphylococcus* spp and other organisms may play an adjunctive role in skin microbiome control for general surgeries. In this study, a decolonization protocol did not show overall benefit in overall SSI reduction in patients undergoing colorectal surgery. However, there was reduction in the risk of superficial infections in patients who did undergo decolonization. Many of our SSIs were treated without identification of a specific pathogen, and thus may have been driven by skin flora, which can be affected by decolonization.

Surface decolonization would not be expected to influence gut flora that are accessed during colorectal procedures, so impact on organ-space infections, for example, as a result of an anastomotic leak, would not be expected. Nevertheless, superficial infections put the patient at risk of longer hospital stays, greater antibiotic exposure, and other postoperative complications that can increase overall postsurgical costs.^10^

Our study had several limitations. First, analyses were cross sectional in nature and the clinical information was gathered from the patients’ medical history, so missing data not obtained perioperatively were not captured. We were not able to include immunosuppression as a variable due to incomplete information on status of immunosuppression in our patient cohort. Also, only 33.7% of our patients received and completed the decolonization procedures. Patient compliance, while documented daily on a checklist, was self-reported. Prior to 2017, decolonization was not a standard process. After the 2017 implementation of decolonization protocols for colorectal surgery, there remained incomplete adherence related to logistical challenges. Possibly, especially prior to standardized protocol in 2017, more complex patients were considered for decolonization because this was based on provider preference. Importantly, even with full compliance, decolonization will not impact all flora associated with SSI, particularly in colorectal surgeries. Because other interventions may have affected SSIs, such as changes in wound-closure strategy over time, this may be a confounder; however, we were not comparing data before and after an intervention time point. Rather, we were considering the entire 5-year period. Lastly, this study was conducted at a single center, and despite 5 years of data, it may be underpowered to adequate assess for differences in SSI rates. Also, the experiences of our patients may not represent colorectal surgery patients in general. For example, in centers with rare occurrence of superficial incisional infections complicating surgeries, the potential benefit of a staphylococcal decolonization protocol that includes mupirocin may not outweigh concerns regarding the development of mupirocin resistance. It is also unclear whether ongoing CHG bathing in the perioperative period, as part of a bundle^11^ or as an isolated intervention, might also reduce superficial wound infections similarly to a full staphylococcal decolonization protocol.

In summary, staphylococcal decolonization may prevent a subset of SSIs in patients undergoing colorectal surgery. In an era of intense competition among healthcare centers for improved surgical outcomes, expansion of decolonization protocols to colorectal surgeries represents a low-risk, low-cost intervention that may prevent superficial wound infections and could be further investigated in other general surgery populations in efforts to optimize perioperative infection prevention.

## Supplementary material

For supplementary material accompanying this paper visit https://doi.org/10.1017/ash.2022.262. click here to view supplementary material
