# Supplementary material for: Staphylococcal decolonization to prevent surgical site infection: Is there a role in colorectal surgery?
Source: Antimicrob Steward Healthc Epidemiol. 2022 Jul 11;2(1):e116. doi: 10.1017/ash.2022.262 (PMC9726530; doi:10.1017/ash.2022.262)
Supplement: Supplementary file 1 [file S2732494X22002625sup001.docx]

**Appendix 1: Decolonization Cohort by Year**

| **Year** | **Decolonized N (%)** | **Not Decolonized N (%)** | **Yearly Case Totals** |
| --- | --- | --- | --- |
| Jul 2015 - Dec 2015 | 25 (29.1) | 61 (70.9) | 86 |
| 2016 | 69 (29.0) | 169 (71.0) | 238 |
| 2017 | 57 (17.5) | 268 (82.5) | 325 |
| 2018 | 99 (30.8) | 222 (69.2) | 321 |
| 2019 | 148 (42.2) | 203 (57.8) | 351 |
| Jan 2020 - Jun 2020 | 170 (47.0) | 192 (53.0) | 362 |
| Study Total | 568 (33.7) | 1115 (66.3) | 1683 |

|  | **Decolonized**  **N=568, N (%)** | **Not Decolonized**  **N=1115, N (%)** | **Odds Ratio (95% CI)** | **p value** |
| --- | --- | --- | --- | --- |
| Overall SSI | 25 (4.4) | 67 (6.0) | 0.72 (0.45,1.15) | 0.17 |
| SWI | 7 (1.2) | 32 (2.9) | 0.42 (0.18,0.96) | 0.04 |
| IAI | 18 (3.2) | 35 (3.1) | 1.01 (0.57,1.80) | 0.97 |

**Appendix 2: Decolonization Status as a Predictor of SSI: Unadjusted**

SSI = surgical site infection, SWI = superficial wound infection, IAI = intra-abdominal infection
